# Effects of Dark Brooders on Behavior and Fearfulness in Layers

**DOI:** 10.3390/ani6010003

**Published:** 2016-01-07

**Authors:** Anja B. Riber, Diego A. Guzman

**Affiliations:** 1 Department of Animal Science, Faculty of Science and Technology, Aarhus University, Blichers Allé 20, P.O. Box 50, Tjele DK-8830, Denmark; 2 Instituto de Investigaciones Biológicas y Tecnológicas (CONICET-UNC) and Instituto de Ciencia y Tecnología de los Alimentos, Facultad de Ciencias Exactas, Físicas y Naturales, Universidad Nacional de Córdoba, Av. Vélez Sarsfield 1611 (5000), Córdoba X5016, Argentina; guzmandiego@hotmail.com

**Keywords:** activity, behavior, brooders, fear, laying hen, novel object, open-field, poultry, tonic immobility, welfare

## Abstract

**Simple Summary:**

Chicks require heat to maintain body temperature during the first weeks after hatch. Heat is normally provided by use of heating lamps or whole-house heating, but an alternative is dark brooders, *i.e.* horizontal heating elements equipped with curtains. The effects of providing layer chicks with dark brooders during the brooding period on behavior and fearfulness were investigated. Brooders resulted in chicks showing less locomotive activity, feather pecking and fleeing. Also, a long-term reduction of fearfulness in brooder birds was found. Results support the suggestion that rearing with dark brooders can be a successful method of reducing or preventing some of the major welfare problems in layers.

**Abstract:**

Chicks require heat to maintain body temperature during the first weeks after hatch. This may be provided by dark brooders; *i.e.*, horizontal heating elements equipped with curtains. The objective was to test effects of rearing layer chicks with dark brooders on time budget and fearfulness. Behavioral observations were performed during the first six weeks of age. Three different fear tests were conducted when the birds were age 3–6, 14–15 and 26–28 weeks. During the first four days, brooder chicks rested more than control chicks whereas they spent less time drinking, feather pecking and on locomotion (*p* ≤ 0.009). On days 16, 23, 30 and 42, brooder chicks spent less time on feather pecking, locomotion and fleeing (*p* ≤ 0.01) whereas foraging and dust bathing occurred more often on day 42 (*p* ≤ 0.032). Brooder birds had shorter durations of tonic immobility at all ages (*p* = 0.0032), moved closer to the novel object at age 15 weeks (*p* < 0.0001), and had shorter latencies to initiate locomotion in the open-field test at age 28 weeks (*p* < 0.0001). Results support the suggestion that dark brooders can be a successful method of reducing or preventing fear and feather pecking in layers.

## 1. Introduction

Chicks require extra heat to maintain body temperature during the first weeks after hatch [1]. In many countries, the extra heat for chicks reared under intensive conditions is provided by heating the entire rearing house. An alternative is to simulate the brooding behavior of a mother hen by using dark brooders. Dark brooders are horizontal heating elements equipped with curtains so that dark and warm areas are created for the chicks to creep under when they are in need of extra heat. The ambient temperature outside the brooders may then be kept at around 18 °C–24 °C [2,3], potentially reducing the energy consumption required to heat the poultry house during rearing of chicks.

Already at a young age, chicks manage to control their body temperature by behavioral thermoregulation. Brooding by a mother hen is usually initiated by chicks approaching the hen, sometimes pecking her breast feathers, and then starting to nuzzle under her [4]. When reared without a broody hen, 1- to 3-day old chicks are capable of choosing the correct arm in a maze or pecking a key to receive warmed air or radiation from a heat lamp [5,6,7]. The brooding behavior of the entire group is not completely synchronized as some chicks enter and leave the brooding hen before others [4,8]. In the red junglefowl, termination of brooding is initiated by the brooded chicks themselves 80% of the time [4]. The remaining 20% of the brooding bouts are ended by the broody hen leaving the brooding posture with chicks still under her [4]. The number of chicks under the broody hen, when she leaves the brooding position, is higher in cold compared to warm conditions [4]. Thus, in one out of five times, the broody hen plays an active role in initiating activity bouts in her chicks affecting more chicks the colder the ambient temperature is. She further encourages the chicks to start feeding by adding gestural and vocal food calling elements to her pecking behavior [9,10,11]. It has been found that brooded layer chicks indeed do perform more feeding and foraging activities than non-brooded chicks [11,12,13].

Rearing chicks with a broody hen during the first weeks of life also has an effect on their emotional and social reactivity later in life. Laying hens reared with a broody hen have been found to be less neophobic at 22 weeks of age than laying hens reared without broody hens as they will go nearer a novel object in their home pens [14]. Likewise, it has been shown that brooded chicks are less fearful than non-brooded chicks in a fear test performed in their home pen where the response to the sudden standing up of a human observer was recorded at age 1–8 weeks [15]. When placed in a very novel situation (*i.e.*, fear tests outside the home pens), 4- to 6-week old brooded layer chicks showed less fear-related behavior than non-brooded chicks [13,16], but later in life this difference disappeared [14,17]. Brooded layer chicks have been shown to be less aggressive than non-brooded chicks [18], and this difference persisted throughout the rearing and laying periods [17]. At 28–29 weeks of age, laying hens that were brooded by a mother hen during rear were more likely to seek proximity to a conspecific than laying hens that were non-brooded during rear [14].

In some aspects, the dark brooders resemble a hen in the brooding posture. Although the brooders, unlike the broody hen, do not interact actively with the chicks, rearing with dark brooders increases the synchrony of activity within the group compared to groups of non-brooded chicks [8]. Previous experiments have shown that rearing layer chicks with dark brooders has a reducing effect on feather pecking behavior during rear and early-lay [3,19], a positive effect on plumage condition during the laying period [2], and a reducing effect on cannibalistic incidences during rear and early lay [3]. In addition to having a preventive effect on feather pecking and cannibalism, the brooders may affect other types of behavior of the birds. For example, caretakers and researchers testing brooders, both in commercial settings and in experimental set-ups, have reported anecdotally that chicks, pullets and adult hens reared with brooders seem to be calmer and less flighty than those reared without brooders [2,20].

Until now, no studies have investigated the effects of dark brooders on time budget and fearfulness in poultry. Some breeders have expressed reluctance against using dark brooders, as they fear an increased number of non- or slow-starter chicks, *i.e.*, chicks that never learn to use brooders efficiently and therefore either do not enter under the brooders when in need of extra heat or do not leave the brooder when in need of food or water. The main objective of the present study was to test short- and long-term effects of rearing layer chicks with dark brooders on time budget and fearfulness. We also aimed to examine whether time budget and fearfulness were affected (a) by the size allowance of the brooder allocated per chick and (b) by raising the brooders for short periods during the first four critical days where chicks have to learn to find food and water. The latter was thought to simulate the active role played by the broody hen in initiating activity bouts. We hypothesized that brooder chicks would be less fearful in the three different fear tests performed (tonic immobility test, open-field test and novel object test) and that the effect would be long-lasting. Also, we predicted that brooder chicks would feather peck less, perform fewer aggressive interactions and show less fleeing than non-brooded chicks. We expected to find more drinking, feeding and foraging during the first four days in the brooder treatments where the brooders were raised for short periods compared to the brooder treatments where the brooders were maintained at the same height during the entire brooding period.

## 2. Experimental Section

### 2.1. Animals and Housing Conditions

Isa Warren layers (*n* = 2254) were obtained from a commercial hatchery (TopÆg Aps, Viborg, Denmark) as newly hatched non-beak-trimmed female chicks and transported to the experimental poultry facility at AU Foulum (Tjele, Denmark). Immediately upon arrival, the chicks were randomly assigned to one of 22 pens located in two different experimental rooms, B and C.

#### 2.1.1. Brooder and Control Groups

In Room B, brooders (one per pen) were used as the primary heat source for the chicks. Upon arrival, chicks were placed under the brooders. The temperature under the brooders was kept constant at 34 °C during the first 3 days and then lowered half a degree each day until 20 °C were reached on day 28. Also, the ambient temperature in Room B was kept at 24 °C on Days 0 and 1, 22 °C on Days 3 and 4, and then kept constant at 20 °C. In room C, no brooders were used, and the ambient temperature in the room was kept at the same temperature schedule as used for the temperature under the brooders. Sixteen groups were housed in Room B and six in Room C. The conditions in the rooms were otherwise exactly the same. The heat sources used for keeping the schedules for the ambient temperature in Rooms B and C were water heated vertical radiators mounted on the outer walls, which is the traditional way to heat poultry houses in Denmark.

#### 2.1.2. The Four Brooder Treatments

Pens in Room B were randomly assigned to one of four treatments that differed in the size of the brooder placed in each pen (Large or Small) and in the management of the height of the brooders (raised for 10 min every 4 hours on Days 1–4 or maintained at the same height during brooding; Movable or Fixed, respectively). Hence four types of brooders/management combinations were tested: LM (Large and Movable), LF (Large and Fixed), SM (Small and Movable) and SF (Small and Fixed). The large brooders measured 120 cm × 60 cm corresponding to an average available area under the brooder of 72 cm^2^ per chick whereas the small brooders measured 90 cm × 60 cm (54 cm^2^/chick). The regular height positioning of the brooders was adjusted only once at 29 days of age to compensate for the growth of the chicks (shifted from 16 cm at the beginning of the experiment to 25 cm on Day 29). All brooders were permanently raised to 2.0 m on Day 41.

#### 2.1.3. Available Resources and Management

Each pen (2 m × 4 m) housed a group of 100–103 chicks, resulting in a stocking density similar to that used for organic pullets and around 2/3 of that used for barn pullets under commercial conditions. Pens were provided with seven automatic water nipples and two feeders (251 cm feeder space, totally). In addition, chicks were fed on paper during the first 4 days. Seventy centimeter high white sheets of hard plastic were attached to the bottom sides of the pens to avoid potential visual contact between individuals from neighboring pens. Bedding was provided in the form of an approximately 5 cm deep layer of wood shavings, and birds were fed a commercial diet (DLG Group, Denmark) according to their rearing stage that was provided *ad libitum*. To enable data collection on time budget from video recordings during the first weeks, where chicks are quite small, we tried to enhance the image quality of the chicks by halving the available floor area using wooden partitions during the first 18 days of the experiment. At Day 18, each pen was provided with two perches (each 2 m long, at 1 m height).

In order to minimize potential undesired minimal differences between rooms/pens influencing the data collection, all groups were reallocated among the 22 available pens in both rooms after completion of the first round of all the fear tests at Day 44 (3 days after the brooders had been permanently raised). This was done in a balanced way so that one group from each of the four brooder treatments and two control groups were housed in Room C and the remaining groups in room B. The allocation of pens within rooms was random, except that a group could not be allocated to its original pen. Time spent daily by caretakers and observers in the two rooms from arrival of the chicks up to the day of reallocation (Day 44) of the groups in the two rooms was approximately the same. Both caretakers and observers noted how much time they spent in Room B and then spent the same amount of time in Room C. This was done in order to avoid differences between treatments in amount of human contact, which potentially could have influenced the fear testing.

At Days 93–94, each pen was provided with six nest boxes (40 cm × 30 cm × 34 cm; W × D × H) and additional perches arranged in a ladder layout providing easy access to the nest boxes. When pullets were 113–114 days old, all groups were reduced to 50 individuals per pen to maintain the stocking density similar to that used for organic hens and around 2/3 of that used for barn hens under commercial conditions during the laying period. Birds removed were mainly those that had been tested in fear tests during 3–6 and 14–15 weeks of age (described below). On Days 118–119 the bedding was removed and replaced by a new layer of approximately 5 cm wood shavings. A computerized system allowed control of light and ventilation, which followed commercial standard practices.

#### 2.1.4. Ethical Note

All procedures involving animals were in accordance with the Danish Ministry of Justice Law No. 382 (10 June 1987) and Acts 333 (19 May 1990), 726 (9 September 1993) and 1016 (12 December 2001). This study is part of a larger project that also evaluates the effects of brooders on welfare and production parameters.

### 2.2. Data Collection

#### 2.2.1. Behavioral Time Budget

Immediately upon arrival and housing, 10 chicks per group were randomly selected and marked on their back with non-toxic, green spray paint (suitable for animals). To ensure further identification, chicks were remarked on Days 7, 14, 21 and 28. Marked chicks were used as focal animals [21] for time budget determination. Originally, the intention was to collect data on activity from video recordings, but even with the temporarily reduced floor area it was impossible to identify the focal animals on the video recordings. Therefore, we decided to do live observations. The live observations were conducted when the chicks were 1, 2, 3, 4, 9, 16, 23, 30, and 42 days of age using instantaneous scan sampling. The observer slowly walked from pen to pen. When in front of a pen (visible to the chicks) the observer stood still for 15 s before recording the behavior of the marked chicks. In order to minimize disturbances from the schedule of raising brooders in the movable treatments, two different behavioral time budget sampling protocols were used depending on the age of the chicks. The first protocol was used for sampling on Days 1, 2, 3 and 4 of age (Phase I) and consisted of three sets of 12 samples each at regular intervals in the periods between 0612 and 0952 h, 1152 and 1532 h and 1812 and 2152 h, respectively (photophase 2–24 h). The second protocol was used on Days 9, 16, 23, 30 and 42 of age (Phase II) and consisted of 27 samples at regular intervals during the period of time between 0612 and 1702 h (photophase 6–18 h). The experimental environment (scheduled changes in the light regime and raising of the brooders), our operational capabilities and Daigle and Siegford’s [22] recommendations regarding time interval between scans were taken into consideration when determining each of these two protocols.

Within each time-point, the number of marked chicks per group performing any of the following 11 behavioral categories was recorded: drinking, feeding, locomotion, foraging, resting, aggression, fleeing, comfort behavior (defined as the chicks stretching its legs and/or wings or wing flapping), dust bathing, exploration and feather pecking. Fleeing was defined as the chick running from frightening stimuli, typically the arrival of the observer, and included possible “freezing” of the chick at the end of the flight, *i.e.*, standing erect and still for a moment until other types of behavior were resumed. Within the brooder groups, resting was split into the following three subcategories: resting outside, under, or upon the brooder. On Day 42, this subdivision was not applicable as the brooders were no longer available for the chicks. Data on time budget were expressed as daily proportions of marked chicks per pen engaged in each of the behavioral categories registered.

#### 2.2.2. Novel Object Test

A Novel Object (NO) test was performed in the home pens at three different developmental stages; Days 43 (chicks), 107 (pullets) and 189 (adult hens) of age. Different objects may elicit different levels of fear, and presenting an object multiple times may result in habituation to the object [23]. Therefore, three different objects were presented in a balanced way between treatments and ages so that one replicate of each of the brooder treatments and two of the control replicates were presented with the same novel object per round of testing. The novel objects used were a red soft drink can, a blue folder and a yellow bucket. To fit this balanced schedule, only 18 pens were tested (six control groups and three groups from each of the four brooder treatments; the same groups were tested across the different test rounds). Each test lasted 5 minutes and began when the object was placed on the floor within an arm’s reach from the door, thus avoiding that a person had to enter the pen. Feed and water were located at least 1 m from the NO.

Data on the birds’ proximity to the novel object were collected from digital video recordings using instantaneous scan sampling at 15, 30, 60, 120, 180, 240 and 300 s after introduction of the novel object. On the obtained pen images, three similar sized zones were drawn on the screen (using reference marks placed in the pens). These zones were delimited in relation to the distance to the novel object: nearest, middle and most distant from the object. For each time-point sampled, the number of birds in each area and the number of birds touching the novel object were counted. Then, those counts were transformed into percentages to enable comparisons between different rounds. Lastly, according to Hocking *et al.*, [23], for each time-point sampled a Mean Score Proximity was calculated using the following calculation:

$$Mean\;Score\;Proximity=\frac{1\times \%Touching+2\times \%Nearest+3\times \%Middle+4\times \%Distant}{100}$$

The position scores ranged from one for the percentage of birds found in the closest proximity to four for the percentage of birds found in the distant area. Thus, a low Mean Score Proximity indicates low avoidance of the novel object.

#### 2.2.3. Open-Field Test

Individual open-field (OF) responses were tested at three different developmental stages (chicks 19–23; pullets 98–106; and adult hens 192–199 days old, from here on referred to as Days 21, 101, and 196) as a measure of fear and exploration [24,25]. Ten naive birds (neither previously tested in OF nor in the tonic immobility test) randomly chosen from each pen were tested at each age. Eight unpainted plywood OF arenas measuring 1.22 m × 1.22 m × 0.81 m (width × depth × height) were located on concrete floor (six in room B and two in room C). The OF arena was divided into 25 squares (24.4 × 24.4 cm) using white markings (chalk). A wire mesh lid was used to prevent potential escapes on Days 101 and 196. Briefly, to begin a test, a bird was caught in her home pen and individually placed in the center of the empty OF arena. Her behavior was video recorded for 10 min by using a closed-circuit television system with a video camera suspended above the OF arena. This arrangement made certain that the experimenter was completely hidden from the bird’s view during testing. Upon completion of the test, birds were marked (using plastic leg bands) before being returned to their respective pens to prevent birds from being reevaluated on following tests or rounds. All birds within a pen were tested before continuing to a new pen. The order of pens tested was random, decided by drawing a number from a pot. This procedure was repeated for each round of testing. From the first 520 s of the video recordings of the test, the following behavioral categories were registered using focal sampling: latency to initiate locomotion and number of lines crossed in the square gridline floor. The number of droppings was counted when 10 min had passed and the OF arena was cleaned after each individual.

#### 2.2.4. Tonic Immobility Test

Tonic Immobility (TI) responses were tested at three different developmental stages (chicks 25–27; pullets 95–98; and adult hens 179–183 days old, from here on referred to as Days 26, 96 and 181) as a measure of fearfulness [25]. Ten naive birds (neither previously tested in OF nor in TI) randomly chosen from each pen were tested at each age. Briefly, the bird to be tested was carefully lifted from its home pen and placed on its back in a wooden cradle situated in the same room. The observer held the bird softly but firm with one hand over the chest and the other hand over the neck/head for 15 s. After releasing the bird, the observer slowly took one step backwards and avoided eye contact with the bird. If the bird remained motionless for 10 s after release, it was deemed to have entered TI. If it moved within 10 s, the procedure was repeated and the number of induction attempts recorded. If TI could not be induced by the third attempt, the bird was deemed to be non-susceptible and a TI duration of 0 s was given. A test ceiling of 10 min was set, and any bird that failed to right itself before this time elapsed was given a maximum score of 600 s, and the test was interrupted. The observer wore similar clothes with a similar color as the caretakers.

The order of pens tested was random, decided by drawing a number from a pot. To prevent birds from being re-tested on following tests or rounds, upon completion of testing time, birds were marked (using plastic leg bands) before being returned to their respective pens. For each TI test performed, the following parameters were registered: the number of inductions, the latency to first head movement (scanning movement), and the duration of TI. During the first round of fear testing, the order of the tests were OF, TI, and NO. During the second and third rounds the order of the tests was TI, OF and NO.

### 2.3. Statistical Analysis

Similar statistical procedures were performed to evaluate the effect of treatments on the expression of each of the behavioral categories registered. Because two different protocols were used for Phase I and II, data from each Phase were analyzed independently. Briefly, daily proportions of marked chicks per pen engaged in each of the behavioral categories were calculated for each day of data collection per treatment and pen. Differences in expression of each of the behavioral categories were determined by a mixed model ANOVA with treatment (LM, LF, SM, SF and C) as fixed effect and days of age (Phase I: 1, 2, 3 and 4; Phase II: 9, 16, 23, 30 and 42) as a repeated measure. Pen was included as a random effect. Due to low occurrences, *i.e.*, large numbers of zeros, the behavioral categories fleeing, aggression, dust bathing, and exploration during Phase I and aggression during Phase II could not be statistically analyzed.

Differences on Mean Score Proximity to the NO were determined by a mixed model ANOVA with treatment (LM, LF, SM, SF and C) as fixed effect and sampling time after introduction of the NO (15, 30, 60, 120, 180, 240 and 300 s) and age at testing (Days 43, 107 and 189) as repeated measures. Pen was included as a random effect. Differences on variables registered during OF and TI tests were determined by a mixed model ANOVA with treatment (LM, LF, SM, SF and C) as fixed effect and age at testing (OF: Days 21, 101, and 196; TI: Days 26, 96, and 181) as repeated measures. Pen was included as a random effect.

In all models used, heteroscedasticity was corrected by specifying the variance structure of the errors whenever necessary. Stepwise reduction of the models was conducted and non-significant interactions were removed from the model. Models were fit using the *nlme* R library through a user-friendly interface implemented in InfoStat [26]. Whenever significant main effects were detected, a *post-hoc* DGC test (hierarchical multiple comparison procedure) was performed [27]. One of the four SM groups had to be removed from all statistical analyses since we discovered the presence of a rooster in the group when the birds were 85 days old; statistical analysis of the NO test was conducted without change as this SM group was not initially selected for testing.

## 3. Results

The results on the time budget for the different treatments are summarized in Table 1 for Phase I and in Table 2 for Phase II. In general, the time budgets for the brooder treatments differed more from the time budget of C than from one another.

During Phase I, there were significant interactions between treatment and age for five behavioral categories. The highest percentages of chicks feeding during Days 1 and 2 were found in C while the lowest percentages were found in SM and SF on Days 2 and 4, respectively (F_12,48_ = 3.78; *p* < 0.001). Additionally, the highest percentage of feeding chicks on Day 4 was found in SM. Fewer chicks rested in C on all days recorded while the highest percentage of chicks resting was found in SM on Day 2 (F_12,48_ = 3.21; *p* = 0.002). The highest percentage of chicks performing comfort behavior was found in C on all days recorded (F_12,48_ = 2.32; *p* = 0.019). No differences between days or treatments were found in the percentage of chicks resting under the brooder, except on Day 2 where a higher percentage of chicks was resting under the brooder in SM (F_9,33_ = 2.88; *p* = 0.012). More variation was found in the percentage of chicks resting outside the brooders with a higher percentage for all treatments on Day 1 compared to Days 3 and 4 (F_9,33_ = 3.14; *p* = 0.007). Additionally, there was a treatment effect for the behavioral categories drinking, locomotion and feather pecking: the highest percentages of chicks drinking (F_4,16_ = 4.83; *p* = 0.009), performing locomotion (F_4,16_ = 21.09; *p* < 0.001) and feather pecking (F_4,16_ = 11.27; *p* < 0.001) were found in C regardless of the age of the chicks. There was an increase in percentage of chicks foraging with increasing age (F_3,48_ = 29.4; *p* < 0.0001; Means ± SE: 1.9 ± 0.2, 2.5 ± 0.2, 3.5 ± 0.3, and 6.9 ± 0.5 on Days 1, 2, 3 and 4, respectively). In Phase I, the behavioral categories aggression, fleeing, dust bathing, and exploration constituted less than 1% of the time budget in all treatments.

During Phase II, there were significant interactions between treatment and age for 11 behavioral categories. A general pattern of decreasing percentages of resting chicks with age was found for all treatments, but the decrease from day to day varied between treatments (F_16,64_ = 3.76; *p* < 0.001). A higher percentage of chicks was resting under the brooders in the treatments with small brooders (SF and SM) on Day 9 (F_9,33_ = 2.18; *p* = 0.049). No differences were found between brooder treatments in percentage of chicks resting outside the brooders during any of the days in Phase II (F_9,33_ = 1.05; *p* = 0.42), but the percentages increased with age (F_3,33_ = 32.53; *p* < 0.001). Likewise, the percentages of chicks resting on the brooders increased with age but faster in the movable brooder treatments (SM and LM; F_9,33_ = 5.77; *p* < 0.001). More chicks were found feeding on the last day (Day 42) compared to the first day (Day 9) of observation in Phase II, and a higher percentage of chicks from the brooder treatments compared to C were feeding on Day 42 (F_16,64_ = 3.88; *p* < 0.001). A similar pattern was found for percentage of chicks foraging (F_16,64_ = 3.13; *p* < 0.001).

**Table 1:** Proportion of layer chicks (%) performing various behavioral categories when 1, 2, 3, and 4 days old. Gray shaded areas indicate no available brooders.

Behavioral measurements (%) ^1^	SF^2^ (Day)				SM^2^ (Day)				LF^2^ (Day)				LM^2^ (Day)				C^2^ (Day)			
	1	2	3	4	1	2	3	4	1	2	3	4	1	2	3	4	1	2	3	4
**Resting**	82.0 ± 1.6 **^b^**	81.8 ± 1.6 **^b^**	79.3 ± 2.3 **^b^**	79.0 ± 2.6 **^b^**	84.0 ± 1.8 **^b^**	88.1 ± 1.9 **^a^**	80.2 ± 2.7 **^b^**	76.1 ± 3.0 **^b^**	83.4 ± 1.6 **^b^**	83.4 ± 1.6 **^b^**	76.2 ± 2.3 **^b^**	73.9 ± 2.6 **^b^**	84.0 ± 1.6 **^b^**	83.3 ± 1.6 **^b^**	78.3 ± 2.3 **^b^**	75.0 ± 2.6 **^b^**	71.3 ± 1.3 **^c^**	64.6 ± 1.3 **^d^**	65.5 ± 1.9 **^d^**	64.6 ± 2.1 **^d^**
**Resting under the brooder**	73.7 ± 0.9 **^b^**	77.0 ± 1.2 **^b^**	75.9 ± 2.5 **^b^**	76.6 ± 2.0 **^b^**	75.4 ± 0.9 **^b^**	84.3 ± 1.0 **^a^**	76.4 ± 3.7 **^b^**	73.7 ± 5.0 **^b^**	74.2 ± 0.5 **^b^**	78.9 ± 0.9 **^b^**	73.9 ± 0.8 **^b^**	71.6 ± 2.7 **^b^**	77.4 ± 2.1 **^b^**	77.6 ± 1.0 **^b^**	74.8 ± 1.9 **^b^**	70.6 ± 2.3 **^b^**				
**Resting outside the brooder**	8.3 ± 1.6 **^a^**	4.7 ± 0.7 **^b^**	3.3 ± 0.8 **^b^**	2.4 ± 0.3 **^c^**	8.6 ± 1.5 **^a^**	3.8 ± 0.6 **^b^**	3.8 ± 0.4 **^b^**	2.4 ± 0.1 **^c^**	9.1 ± 1.0 **^a^**	4.4 ± 0.9 **^b^**	2.3 ± 0.5 **^c^**	2.3 ± 0.2 **^c^**	6.5 ± 0.6 **^a^**	5.6 ± 0.9 **^a^**	3.6 ± 0.6 **^b^**	4.5 ± 0.3 **^b^**				
**Feeding**	5.6 ± 0.3 **^c^**	6.0 ± 1.0 **^c^**	7.0 ± 1.6 **^c^**	4.0 ± 0.7 **^d^**	5.2 ± 0.4 **^c^**	3.7 ± 0.3 **^d^**	5.1 ± 0.6 **^c^**	8.8 ± 2.6 **^b^**	5.5 ± 0.5 **^c^**	6.0 ± 0.5 **^c^**	9.1 ± 0.6 **^b^**	6.4 ± 1.1 **^c^**	6.5 ± 0.9 **^c^**	6.0 ± 0.5 **^c^**	7.6 ± 1.7 **^c^**	6.5 ± 0.9 **^c^**	8.7 ± 0.5 **^b^**	12.7 ± 1.1 **^a^**	10.4 ± 1.4 **^b^**	6.4 ± 0.8 **^c^**
**Comfort**	0.5 ± 0.2 **^c^**	0.0 ± 0.2 **^c^**	1.1 ± 0.2 **^b^**	1.4 ± 0.2 **^b^**	0.2 ± 0.1 **^c^**	0.0 ± 0.1 **^c^**	0.6 ± 0.1 **^c^**	1.4 ± 0.1 **^b^**	0.1 ± 0.2 **^c^**	0.1 ± 0.2 **^c^**	1.5 ± 0.2 **^b^**	1.9 ± 0.2 **^b^**	0.4 ± 0.3 **^c^**	0.1 ± 0.3 **^c^**	1.1 ± 0.3 **^b^**	1.5 ± 0.3 **^b^**	1.3 ± 0.5 **^b^**	2.2 ± 0.5 **^b^**	4.3 ± 0.5 **^a^**	5.0 ± 0.5 **^a^**
**Drinking**	3.1 ± 0.3 **^b^**				3.0 ± 0.4 **^b^**				3.0 ± 0.4 **^b^**				3.5 ± 0.4 **^b^**				4.6 ± 0.3 **^a^**			
**Locomotion**	6.1 ± 0.5 **^b^**				5.5 ± 0.6 **^b^**				5.8 ± 0.3 **^b^**				4.8 ± 0.3 **^b^**				9.7 ± 0.4 **^a^**			
**Feather pecking**	0.1 ± 0.4 **^b^**				0.1 ± 0.4 **^b^**				0.1 ± 0.4 **^b^**				0.2 ± 0.1 **^b^**				0.9 ± 0.1 **^a^**			

^1^ The values represent means ± SE; (%) = Daily average percentages of focal chicks performing a particular behavior (calculated from 36 observations per group per day); ^2^ Treatments = SF: small fixed brooder; SM: small movable brooder; LF: large fixed brooder; LM: Large movable brooder; C: Control; **^a–d^** Values within a behavioral category with no common letters differ significantly (*p* < 0.05).

**Table 2:** Proportion of layers (%) performing various behavioral categories when 9, 16, 23, 30, and 42 days old. Gray shaded areas indicate no available brooders.

Behavioral measurements (%) ^1^	SF^2^ (Day)					SM^2^ (Day)					LF^2^ (Day)					LM^2^ (Day)					C^2^ (Day)				
	9	16	23	30	42	9	16	23	30	42	9	16	23	30	42	9	16	23	30	42	9	16	23	30	42
**Resting**	69.6 ± 1.2 **^a^**	55.9 ± 2.3 **^c^**	49.6 ± 2.3 **^c^**	48.9 ± 2.3 **^c^**	36.1 ± 1.3 **^e^**	73.9 ± 2.8 **^a^**	58.4 ± 2.2 **^c^**	50.7 ± 2.9 **^c^**	43.7 ± 1.5 **^d^**	38.6 ± 1.4 **^e^**	64.8 ± 1.7 **^b^**	53.2 ± 1.2 **^c^**	46.7 ± 3.9 **^d^**	44.8 ± 2.9 **^d^**	37.4 ± 0.9 **^e^**	64.9 ± 2.0 **^b^**	58.1 ± 1.4 **^c^**	53.2 ± 2.2 **^c^**	45.9 ± 2.1 **^d^**	40.4 ± 1.7 **^d^**	62,9 ± 1.5 **^b^**	46.7 ± 1.7 **^d^**	42.0 ± 1.9 **^d^**	41.9 ± 2.0 **^d^**	38.1 ± 1.2 **^e^**
**Resting under the brooder**	61.5 ± 2.0 **^a^**	41.3 ± 2.0 **^c^**	35.6 ± 2.7 **^d^**	33.3 ± 1.9 **^d^**		65.6 ± 2.3 **^a^**	42.2 ± 2.3 **^c^**	37.4 ± 3.2 **^d^**	28.5 ± 2.2 **^d^**		56.3 ± 2.0 **^b^**	41.4 ± 2.0 **^c^**	37.7 ± 2.7 **^d^**	32.0 ± 1.9 **^d^**		56.3 ± 2.0 **^b^**	41.4 ± 2.0 **^c^**	37.7 ± 2.7 **^d^**	32.0 ± 1.9 **^d^**						
**Resting outside the brooder**	8.1 ± 0.1 **^a^**	12.5 ± 1.6 **^a^**	14.0 ± 1.6 **^a^**	15.5 ± 1.4 **^a^**		7.4 ± 1.2 **^a^**	16.1 ± 1.8 **^a^**	13.3 ± 1.8 **^a^**	15.1 ± 1.6 **^a^**		8.6 ± 1.0 **^a^**	12.5 ± 1.6 **^a^**	12.7 ± 1.6 **^a^**	16.0 ± 1.4 **^a^**		8.6 ± 1.0 **^a^**	16.7 ± 1.6 **^a^**	15.5 ± 1.6 **^a^**	13.9 ± 1.4 **^a^**						
**Resting on the brooder**	0.7 ± 0.4 **^c^**	10.3 ± 1.9 **^b^**	12.6 ± 0.8 **^b^**	15.2 ± 1.2 **^a^**		0.1 ± 0.1 **^c^**	14.7 ± 2.3 **^a^**	12.3 ± 0.1 **^b^**	16.3 ± 2.2 **^a^**		2.1 ± 1.5 **^c^**	9.9 ± 0.8 **^b^**	12.8 ± 0.6 **^b^**	14.8 ± 1.7 **^a^**		3.8 ± 2.6 **^c^**	17.3 ± 0.8 **^a^**	10.2 ± 0.9 **^b^**	12.9 ± 2.2 **^b^**						
**Feeding**	5.8 ± 1.0 **^c^**	9.1 ± 0.8 **^b^**	11.9 ± 0.9 **^a^**	11.8 ± 1.4 **^a^**	12.2 ± 0.5 **^a^**	5.0 ± 0.6 **^c^**	11.3 ± 1.0 **^a^**	13.6 ± 0.5 **^a^**	11.9 ± 1.9 **^a^**	14.9 ± 2.3 **^a^**	8.0 ± 1.5 **^b^**	9.5 ± 0.5 **^b^**	13.2 ± 1.0 **^a^**	15.4 ± 0.9 **^a^**	13.6 ± 2.0 **^a^**	5.5 ± 0.6 **^c^**	10.2 ± 1.2 **^a^**	14.3 ± 1.1 **^a^**	12.2 ± 1.0 **^a^**	12.1 ± 0.8 **^a^**	6.3 ± 0.8 **^c^**	11.7 ± 1.2 **^a^**	14.3 ± 1.9 **^a^**	10.8 ± 0.9 **^a^**	9.2 ± 0.6 **^b^**
**Foraging**	10.4 ± 1.3 **^c^**	11.2 ± 1.1 **^c^**	12.3 ± 0.7 **^a^**	12.9 ± 1.0 **^c^**	18.6 ± 1.0 **^a^**	6.6 ± 0.2 **^e^**	10.3 ± 0.6 **^c^**	12.6 ± 0.8 **^c^**	12.2 ± 0.9 **^c^**	15.9 ± 1.3 **^b^**	8.3 ± 1.0 **^d^**	14.4 ± 1.2 **^b^**	12.9 ± 1.0 **^c^**	13.1 ± 0.8 **^c^**	14.6 ± 0.9 **^b^**	9.8 ± 1.2 **^c^**	11.8 ± 0.5 **^c^**	10.0 ± 0.9 **^c^**	16.0 ± 2.2 **^b^**	14.9 ± 0.7 **^b^**	8.6 ± 0.5 **^d^**	10.6 ± 0.8 **^c^**	10.9 ± 0.5 **^c^**	11.4 ± 1.2 **^c^**	9.7 ± 1.0 **^c^**
**Comfort**	1.8 ± 0.3 **^c^**	2.9 ± 0.7 **^b^**	4.7 ± 0.1 **^b^**	4.4 ± 0.7 **^b^**	7.4 ± 0.9 **^a^**	1.5 ± 0.2 **^c^**	2.2 ± 0.7 **^c^**	3.8 ± 1.5 **^b^**	6.0 ± 0.9 **^b^**	5.9 ± 1.5 **^b^**	1.5 ± 0.5 **^c^**	2.7 ± 1.0 **^b^**	3.0 ± 0.3 **^b^**	5.2 ± 0.5 **^b^**	7.6 ± 0.8 **^a^**	1.8 ± 0.3 **^c^**	2.3 ± 0.2 **^c^**	3.1 ± 0.7 **^b^**	4.4 ± 0.9 **^b^**	7.0 ± 1.6 **^a^**	3.4 ± 0.6 **^b^**	3.6 ± 0.7 **^b^**	3.8 ± 0.4 **^b^**	4.4 ± 0.6 **^b^**	3.8 ± 0.5 **^b^**
**Drinking**	2.7 ± 0.8 **^b^**	3.1 ± 0.3 **^b^**	3.6 ± 0.8 **^b^**	4.2 ± 0.8 **^b^**	5.8 ± 0.3 **^a^**	1.9 ± 0.9 **^b^**	2.4 ± 0.4 **^b^**	4.6 ± 0.3 **^b^**	5.9 ± 0.3 **^a^**	3.5 ± 0.7 **^b^**	2.8 ± 0.4 **^b^**	3.1 ± 0.6 **^b^**	4.1 ± 0.5 **^b^**	3.6 ± 0.3 **^b^**	4.2 ± 0.4 **^b^**	2.9 ± 0.5 **^b^**	2.6 ± 0.3 **^b^**	3.5 ± 0.5 **^b^**	4.1 ± 0.8 **^b^**	4.1 ± 0.5 **^b^**	2.8 ± 0.4 **^b^**	3.3 ± 0.9 **^b^**	3.3 ± 0.3 **^b^**	2.8 ± 0.5 **^b^**	3.7 ± 0.5 **^b^**
**Locomotion**	8.7 ± 0.6 **^e^**	13.8 ± 1.5 **^d^**	14.3 ± 2.0 **^d^**	10.1 ± 1.0 **^d^**	14.9 ± 1.1 **^d^**	11.0 ± 1.5 **^d^**	12.2 ± 1.7 **^d^**	11.0 ± 0.8 **^d^**	11.3 ± 0.8 **^d^**	15.6 ± 1.2 **^d^**	12.9 ± 2.8 **^d^**	13.7 ± 0.3 **^d^**	14.2 ± 2.1 **^d^**	12.8 ± 0.8 **^d^**	17.9 ± 0.9 **^c^**	12.8 ± 1.5 **^d^**	12.7 ± 0.3 **^d^**	12.1 ± 1.0 **^d^**	11.2 ± 0.9 **^d^**	15.2 ± 1.4 **^d^**	13.2 ± 1.1 **^d^**	18.7 ± 1.2 **^c^**	19.1 ± 1.2 **^c^**	23.2 ± 1.3 **^b^**	27.9 ± 0.9 **^a^**
**Feather pecking**	0.4 ± 0.2 **^b^**	1.6 ± 0.2 **^b^**	1.2 ± 0.6 **^b^**	1.5 ± 0.6 **^b^**	1.0 ± 0.5 **^b^**	0.1 ± 0.1 **^c^**	1.3 ± 0.6 **^b^**	0.9 ± 0.4 **^b^**	0.4 ± 0.2 **^b^**	1.4 ± 0.6 **^b^**	0.0 ± 0.1 **^c^**	0.7 ± 0.3 **^b^**	1.1 ± 0.3 **^b^**	0.9 ± 0.7 **^b^**	1.4 ± 0.8 **^b^**	0.0 ± 0.1 **^c^**	0.6 ± 0.2 **^b^**	0.7 ± 0.4 **^b^**	0.7 ± 0.2 **^b^**	1.0 ± 0.3 **^b^**	1.2 ± 0.1 **^b^**	2.6 ± 0.6 **^a^**	3.2 ± 0.8 **^a^**	2.5 ± 0.7 **^a^**	2.5 ± 0.3 **^a^**
**Fleeing**	0.1 ± 0.2 **^c^**	0.7 ± 0.2 **^c^**	0.3 ± 0.2 **^c^**	0.2 ± 0.2 **^c^**	0.3 ± 0.2 **^c^**	0.1 ± 0.2 **^c^**	0.8 ± 0.2 **^c^**	0.0 ± 0.2 **^c^**	0.3 ± 0.2 **^c^**	0.6 ± 0.2 **^c^**	0.2 ± 0.3 **^c^**	0.5 ± 0.3 **^c^**	0.3 ± 0.3 **^c^**	0.6 ± 0.3 **^c^**	0.5 ± 0.3 **^c^**	0.4 ± 0.2 **^c^**	0.8 ± 0.2 **^c^**	0.0 ± 0.2 **^c^**	0.3 ± 0.2 **^c^**	0.4 ± 0.2 **^c^**	0.8 ± 0.4 **^c^**	1.6 ± 0.4 **^b^**	2.1 ± 0.4 **^b^**	1.7 ± 0.4 **^b^**	3.5 ± 0.4 **^a^**
**Exploration**	0.5 ± 0.2 **^b^**	1.5 ± 0.4 **^b^**	1.9 ± 0.5 **^b^**	5.1 ± 0.5 **^a^**	2.5 ± 0.9 **^b^**	0.4 ± 0.2 **^b^**	0.9 ± 0.5 **^b^**	2.5 ± 0.6 **^b^**	7.4 ± 1.6 **^a^**	3.5 ± 0.7 **^a^**	1.0 ± 0.4 **^b^**	1.7 ± 0.4 **^b^**	3.9 ± 1.3 **^a^**	3.1 ± 1.1 **^a^**	2.1 ± 0.7 **^b^**	1.0 ± 0.3 **^b^**	0.6 ± 0.1 **^c^**	2.3 ± 0.6 **^b^**	4.5 ± 1.0 **^a^**	3.9 ± 0.8 **^a^**	0.6 ± 0.3 **^b^**	0.8 ± 0.3 **^b^**	0.7 ± 0.3 **^b^**	0.8 ± 0.2 **^b^**	1.3 ± 0.4 **^b^**
**Dustbathing**	0.0 ± 0.2 **^b^**	0.1 ± 0.2 **^b^**	0.1 ± 0.2 **^b^**	0.7 ± 0.2 **^a^**	0.9 ± 0.2 **^a^**	0.1 ± 0.2 **^b^**	0.1 ± 0.2 **^b^**	0.3 ± 0.2 **^b^**	0.9 ± 0.2 **^a^**	0.1 ± 0.2 **^b^**	0.4 ± 0.2 **^b^**	0.5 ± 0.2 **^a^**	0.5 ± 0.2 **^a^**	0.3 ± 0.2 **^b^**	0.6 ± 0.2 **^a^**	0.6 ± 0.2 **^a^**	0.2 ± 0.2 **^b^**	0.6 ± 0.2 **^a^**	0.7 ± 0.2 **^a^**	1.0 ± 0.2 **^a^**	0.1 ± 0.1 **^b^**	0.4 ± 0.1 **^b^**	0.3 ± 0.1 **^b^**	0.3 ± 0.1 **^b^**	0.4 ± 0.1 **^b^**

^1^ The values represent means ± SE; (%) = Daily average percentages of focal chicks performing a particular behavior (calculated from 27 observations per group per day); ^2^ Treatments = SF: small fixed brooder; SM: small movable brooder; LF: large fixed brooder; LM: Large movable brooder; C: Control. **^a–d^** Values within a behavioral category with no common letters differ significantly (*p* < 0.05).

In the brooder treatments there was an increase in percentage of chicks performing comfort behavior with increasing age whereas no difference was found between ages in C (F_16,64_ = 3.21; *p* < 0.001). A higher percentage of chicks from the brooder treatments (except SM) compared to C performed comfort behavior on Day 42 (F_16,64_ = 3.21; *p* < 0.001). No differences were found between treatments in percentage of chicks drinking during any of the days in Phase II, except on Days 30 and 42 where SM and SF, respectively, performed more drinking behavior (F_16,64_ = 5.64; *p* < 0.001). The percentage of chicks engaged in locomotion remained stable (with minor exceptions) with age in the brooder treatments and with no difference between treatments whereas a steady increase with age occurred in C, resulting in almost twice as many chicks engaged in locomotion in C compared to any of the brooder treatments at 42 days of age (F_16,64_ = 3.52; *p* < 0.001). The percentage of chicks engaged in feather pecking was higher in C (F_4,16_ = 9.52; *p* < 0.001). Fleeing was continuously rare in the brooder treatments whereas a steady increase with age was found in C (F_16,64_ = 2.28; *p* = 0.01). Exploratory behavior was generally low in C but varied more with age in the brooder treatments (F_16,64_ = 5.11; *p* < 0.001). Finally, dust bathing occurred at low frequencies in all treatments but was rarest in C (F_16,64_ = 1.94; *p* = 0.032). Aggression constituted less than 1% of the time budget in all treatments.

**Figure 1:**
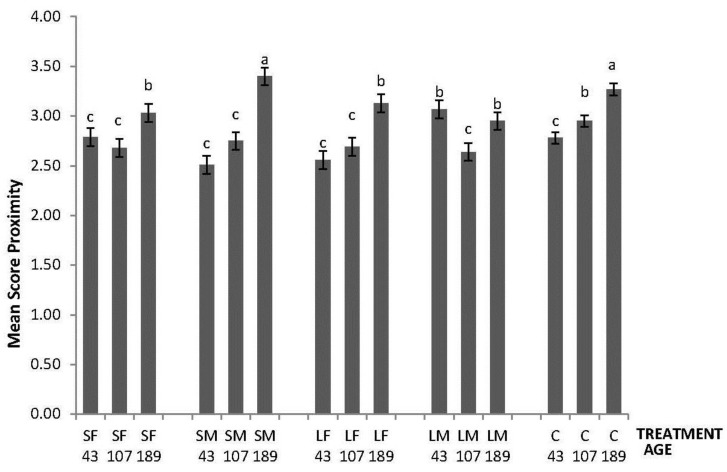
Novel Object test response depicted as Mean Score Proximity of 43, 107 and 189 days old layers from the control and four brooder treatments. Bars with no common letters differ significantly (mixed model ANOVA: *p* < 0.05). Treatments = SF: small fixed brooder; SM: small movable brooder; LF: large fixed brooder; LM: Large movable brooder; C: Control.

In the NO test, an effect of the interaction between treatment and age was found (F_8,260_ = 9.60; *p* < 0.0001); the highest values of Mean Score Proximity to the novel object were obtained by LM on Day 43, by C on day 107, and by SM and C on day 189 (Figure 1).

**Figure 2:**
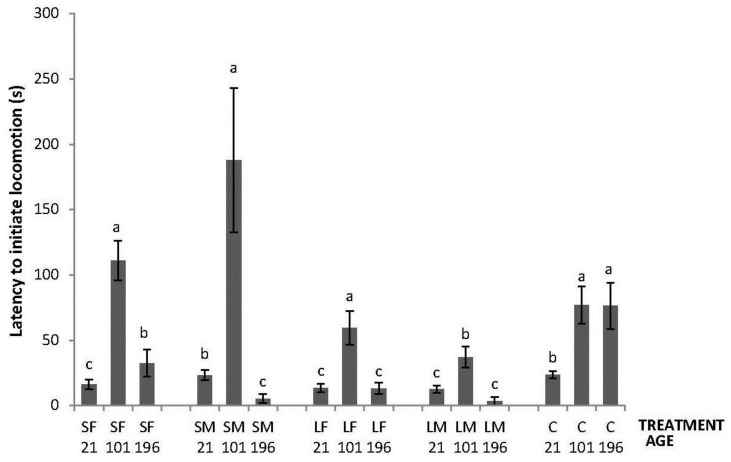
Latency to initiate locomotion in a 520 s open-field test by 21, 101 and 196 days old layers from the control and four brooder treatments. Bars with no common letters differ significantly (mixed model ANOVA: *p* < 0.05). Treatments = SF: small fixed brooder; SM: small movable brooder; LF: large fixed brooder; LM: Large movable brooder; C: Control.

**Figure 3:**
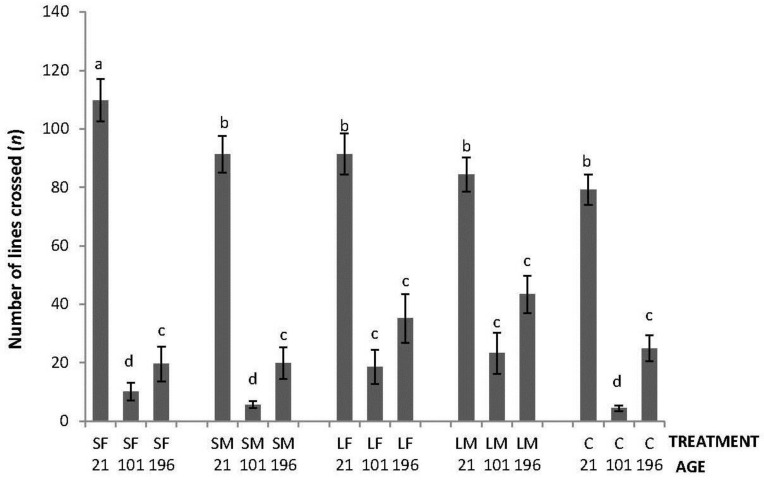
Number of lines crossed in a square gridline floor in a 520 s open-field test by 21, 101 and 196 days old layers from the control and four brooder treatments. Bars with no common letters differ significantly (mixed model ANOVA: *p* < 0.05). Treatments = SF: small fixed brooder; SM: small movable brooder; LF: large fixed brooder; LM: Large movable brooder; C: Control.

**Figure 4:**
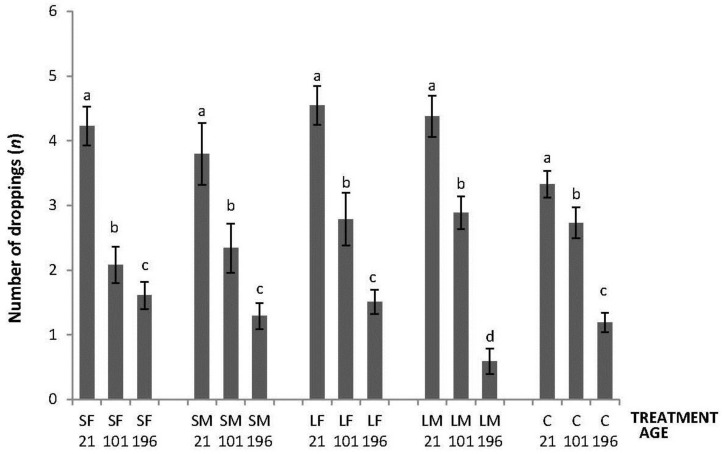
Number of droppings made in 600 s during an OF test by 21, 101, and 196 days old layers from the control and four brooder treatments. Bars with no common letters differ significantly (mixed model ANOVA: *p* < 0.05). Treatments = SF: small fixed brooder; SM: small movable brooder; LF: large fixed brooder; LM: Large movable brooder; C: Control.

Regarding the OF tests performed, there was an interaction between treatment and age in latency to initiate locomotion (F_8,447_ = 5.76; *p* < 0.0001); the highest latencies were obtained by SM and C on Day 21, by SF, SM, LF and C on Days 101, and by C on Day 196 (Figure 2). An interaction between treatment and age was also found in number of lines crossed (F_8,447_ = 2.42; *p* = 0.015). During the OF test on Day 21, only SF differed from the other treatments having a higher number of line crossings. At 101 days of age, more variation was found with a lower number of line crossings by birds from SF, SM and C. Finally, at 196 days of age, no differences between treatments were found (Figure 3). Fewer droppings were produced by LM on Day 196, but otherwise no difference was found between treatments (Figure 4; treatment × age; F_8,447_ = 3.47; *p* = 0.0007).

In the TI test, there was an interaction between treatment and age on number of inductions (F_8,603_ = 2.43; *p* = 0.0138); fewer inductions were necessary to induce TI in C on Days 96 and 181 than in the brooder treatments, whereas no difference was found on Day 26 (Table 3). For latency to first head movement and duration of TI, there was a treatment effect where the highest values for both variables were obtained by C regardless of the age by which the birds were tested (latency: F_4,16_ = 7.58; *p* = 0.0013; duration: F_4,16_ = 6.25; *p* = 0.0032).

**Table 3:** Tonic immobility responses of layers when 26, 96 and 181 days old.

Behavioral measurements ^1^	SF^2^ (Days)			SM^2^ (Days)			LF^2^ (Days)			LM^2^ (Days)			C^2^ (Days)		
	26	96	181	26	96	181	26	96	181	26	96	181	26	96	181
**Inductions (*n*)**	2.4 ± 0.2 **^a^**	2.6 ± 0.2 **^a^**	2.3 ± 0.2 **^a^**	2.6 ± 0.2 **^a^**	2.6 ± 0.2 **^a^**	1.5 ± 0.2 **^b^**	2.7 ± 0.2 **^a^**	2.5 ± 0.2 **^a^**	2.1 ± 0.2 **^a^**	2.6 ± 0.2 **^a^**	2.5 ± 0.2 **^a^**	2.1 ± 0.2 **^a^**	2.1 ± 0.2 **^a^**	1.8 ± 0.1 **^b^**	1.9 ± 0.1 **^b^**
**Latency to first head movement (s)**	69.1 ± 10.0 **^a^**			57.0 ± 10.9 **^a^**			49.3 ± 6.4 **^a^**			80.7 ± 11.4 **^a^**			116.0 ± 10.8 **^b^**		
**TI duration (s)**	105.3 ± 13.2 **^a^**			93.2 ± 12.5 **^a^**			76.6 ± 10.1 **^a^**			112.6 ± 13.0 **^a^**			154.9 ± 12.5 **^b^**		

^1^ The values represent means ± SE; (*n*) = number; (s) = seconds; ^2^ Treatments = SF: small fixed brooder; SM: small movable brooder; LF: large fixed brooder; LM: Large movable brooder; C: Control; **^a–b^** Values within rows with no common letters differ significantly (*p* < 0.05).

## 4. Discussion

Chicks reared with brooders quickly learned to use the brooders for thermoregulation and to leave the brooders for performance of active, including maintenance, behavior. The main objective of the present study was to test short- and long-term effects of rearing layer chicks with dark brooders on time budget and fearfulness, and it is clear from the results that rearing layer chicks with brooders affects their time budget and fearfulness when compared with chicks reared without brooders. We also aimed to examine whether time budget and fearfulness were affected by the type of brooder, and we found minor differences between brooder treatments although results were not as clear and consistent as when comparing birds reared either with or without brooders.

### 4.1. Effects of Dark Brooders on Time Budget: Control vs. Brooder Chicks

We predicted that brooder chicks would feather peck less than control chicks, as previous studies have shown that rearing layer chicks with dark brooders has reducing effects on feather pecking behavior during rear and early-lay [3,19]. Riber [28] hypothesized that the positive effects on development of feather pecking were due to the brooders having a separating effect on active and inactive chicks during the sensitive period for learning about food and appropriate dust bathing material. The sensitive period for recognition of food and dust bathing material is imbedded in the first 10 days of life [29,30,31,32]. During this period, molting is very limited, thus the main source of feathers is the plumage of pen-mates. Consequently, avoiding mixing chicks engaged in foraging or dust bathing activities with inactive chicks lying or sitting on the ground will result in fewer ground pecks misdirected to feathers of conspecifics, and as a result, development of feather pecking will be impeded. Indeed, we found that brooder chicks performed less feather pecking behavior during the first 1–4 days of age (Phase I) and that this reduction in feather pecking behavior persisted throughout Phase II (Days 9, 16, 23, 30 and 42 of age).

We also hypothesized that brooder chicks would show less fleeing than non-brooded chicks. During Phase I, observations of fleeing were too infrequent to be analyzed statistically, but in Phase II, the results on fleeing supported our hypothesis: Brooder chicks showed less fleeing than the control chicks, revealing a lower underlying fearfulness [33]. One may argue that the chicks under the brooders could not perceive the arrival of the observer and, therefore, they did not react to that with fleeing, thereby skewing the results. However, the brooders were removed prior to the last day of observation in Phase II (Day 42) without causing an increase in fleeing on Day 42. The lower underlying fearfulness in brooder chicks was also supported by the results from the fear tests (discussed in 4.3.).

Finally, we predicted that brooder chicks would perform fewer aggressive interactions than control chicks as this has been shown to be the case in brooded chicks compared to non-brooded chicks [17,18]. However, the hypothesis could not be proven due to infrequent observations of aggressive interactions in all treatments, both in Phase I and II.

Brooder chicks spent less time on comfort behavior compared to control chicks during the brooding period (Days 1, 2, 3, 4, 9, 16, 23 and 30). However, on the last observation day (Day 42) where the brooder chicks no longer had access to brooders, they showed more comfort behavior than the control chicks (except chicks from the brooder treatment SM). Comfort behavior was defined as stretching legs and/or wings in addition to wing flapping. Often, these types of behavior occur at the start of renewed activity after a period of rest [34]. We therefore suggest that, in addition to resting, some comfort behavior may have been performed under the brooders and that the lower frequencies found in the brooder groups are an artefact of the impeded observations of behavior under the brooders. As the defined behavioral elements of comfort behavior are quickly performed time-wise, the impact on the estimated time spent resting is thought to be negligible.

#### 4.1.1. Phase I

Overall, a lower level of activity was found during Days 1–4 of age (Phase I) in brooder chicks compared to control chicks; e.g., control chicks spent almost twice as much time on feeding and locomotion compared to the brooder chicks. The brooder chicks spent more time resting, including the time spent under the brooder, where they were assumed to be resting. From what was possible to observe, chicks settled down and rested when entering under the brooder, probably promoted by the darkness and warmth. Similar observations were done in a previous study using brooders [3]. Previous studies have shown limited activity in chicks during the dark periods [35,36,37], unless the difference in light intensity between the light and dark periods is minimal [36,37]. The increased time spent resting in the brooder chicks may be explained by the chicks staying under the brooders to maintain the normal body temperature. In a study of red jungle fowl, Sherry [4] found that the brooding time of 2–5 day-old chicks was 40%–50% longer in conditions with an ambient temperature of 19 °C compared to 28 °C. Behavioral thermoregulation may also at least partly explain the increased time spent on locomotion in control chicks. Control chicks may have been searching for areas with a microclimate within the pen matching their needs at the time. Other explanations of the increased time spent on locomotion in control chicks may be more disturbances of sleep and searches for places to hide [38].

The increased time spent resting in brooder chicks was taken from time spent on locomotion, feeding and drinking. The reduced time spent drinking may be explained by the presence of the brooders allowing the chicks to thermoregulate more efficiently which would result in less respiratory evaporation [39]. A reduced need for water intake may also result from more rest and less locomotion. Higher humidity reduces the respiratory evaporation from the chicks and thus the need for water consumption [40,41]. Not only the temperature but also the humidity under the dark brooders differed from the surroundings. During Phase I, a lower humidity was found under the brooders compared to outside the brooders, but it was still higher than the humidity in the control room (data not shown). Finally, the lower water consumption could also be directly related to reduced food intake, but although brooder chicks spent less time feeding during the first 2–3 days of age, the amount consumed did not differ between treatments [42]. Malleau *et al.* [35] reared layer chicks on two different lighting programs: continuous lighting from 0400–2320 h *versus* alternating light and dark periods of 40 min duration during the same hours as the continuous light was provided in the other treatment group. They found that although the amount of time spent on active behavior decreased, and the amount of time spent resting increased during Days 1 and 4 in the alternating light/dark treatment, no difference was found on feed intake during the period 1–4 days of age. Chicks may, therefore, compensate for the reduced time spent feeding by an increased rate of feed intake.

#### 4.1.2. Phase II

Whereas time spent on feather pecking, locomotion and fleeing remained stable during Days 9, 16, 23, 30 and 42 of age (Phase II) in brooder chicks, these behavioral categories occurred more frequently (except on Day 9) and continued to increase in frequency in control chicks during Phase II. The increase in feather pecking in control chicks is likely to have been linked to their lower level of foraging and dust bathing activities. Feather pecking has been shown to be negatively correlated with ground pecking [43,44,45,46,47,48]. Ground pecking is part of foraging behavior, and it is also an important behavioral element of dust bathing [49,50]. It seems likely that from Day 16, the higher occurrence of locomotion was associated with the increase in feather pecking which also may have instigated the increased emotional reactivity of the chicks making them more prone to show fleeing. Previous studies have found an association between anxiety and feather pecking. For example, Hansen [51] compared laying hens in different housing systems and found that hens in a tiered wire floor aviary system expressed more feather pecking and were more restless, measured as the number of transitions between activities, than hens in other types of aviary systems. The laying hens from the tiered wire floor aviary system also spent more time on locomotion, thus expressing the same positive association between locomotion and feather pecking as found in the present study. Other studies have found chicks showing high anxiety in an open-field test to have stronger tendencies to perform feather pecking [52,53]. Higher levels of locomotion, feather pecking and fleeing can be expected to cause more disturbances of rest. However, the amount of resting in control chicks only seemed to be affected on Day 16 where control chicks rested less than brooder chicks. O’Connor [54] reported that laying hens spent more time resting when sleep was disturbed by continuous high background noise. The apparent discrepancy in reaction to disturbances of rest found between the present study and the study by O’Connor may be attributed to the fact that the sleeping patterns are likely to differ between adult hens and chicks [35]. The factors causing the disturbances differ as well, which may also have an impact on the effect.

### 4.2. Effects of Dark Brooders on Time Budget: Differences between the Brooder Treatments

We expected to find more drinking, feeding and foraging during Phase I in the brooder treatments where the brooders were raised for short periods during the first four days, compared to the brooder treatments, where the brooders were maintained at the same height during the entire brooding period. However, no such differences were found. Thus, raising the brooders in regular intervals during the first days did not turn out to be effective in stimulating maintenance activities (*i.e*., drinking and feeding) in the chicks. No clear trends in the differences in activity were found between the different brooder treatments, except that on Day 9, a higher percentage of chicks were resting under the brooders in the two treatments with a smaller available area under the brooder per chick. This observation may indicate that space was starting to become limited, forcing chicks closer to the borders of the brooders where the temperature was not as high as in the middle, *i.e.*, chicks had to stay under the brooders longer to maintain normal body temperature. However, this speculation was not supported by the observations on percentage of chicks resting outside the brooders, as we did not find any clear differences between brooder treatments on this behavioral category that could indicate competition over space under the brooders.

### 4.3. Effect of Brooders on Fearfulness: Control vs. Brooder Birds

Our hypothesis that birds from the brooder treatments would be less fearful was supported as the results from the TI test clearly showed that birds from the brooder treatments were less fearful, irrespective of which parameter was measured and the age of the birds when tested (with the exception of minor differences in the number of inductions). Also, the results from the NO and OF tests supported the hypothesis, as the overall outcome was that control birds were either the most fearful or among the most fearful birds during the fear tests conducted when the birds were either pullets or adults. The reducing effect on fearfulness of brooders, when the birds were chicks, is similar to what has been found in chicks brooded by mother hens [13]. However, the effect of broody hens on fearfulness has been found to disappear with age when fear tests outside the home pens have been conducted [14,17]. In contrast, we found an increasing discrepancy with age between brooder and control birds in fearfulness with control birds being most fearful. Thus, our hypothesis that the reducing effect of dark brooders would be long-lasting was supported as well. We speculate whether the long-lasting effects on fearfulness of brooders, unlike broody hens, may be associated with the exclusion of emotionality of the brooding element. It has been shown that the fearfulness of the mother hen herself affects the fearfulness of her chicks complicating investigations of fearfulness in brooded chicks [55,56,57]. This factor is omitted when using dark brooders during the brooding phase. Furthermore, most studies show that being brooded in the brooding period by a mother hen, in contrast to dark brooders, does not have a reducing effect on feather pecking activity in chicks, pullets, or young adult hens [15,58]. In contrast, we found a reducing effect of brooders on feather pecking behavior during the observation period, *i.e.*, the first six weeks of age. Feather pecking is known to be positively associated with fearfulness in layers [47,59,60]. Older studies suggest that the pecked birds become more fearful due to the discomfort and pain experienced when being pecked [61,62]. A more recent study has demonstrated that fearful birds have a higher risk of developing severe feather pecking later in life [53]. We therefore suggest that the increase in fearfulness in control birds compared to brooder birds, occurring between the first and second fear tests (chicks and pullets, respectively), may at least partly be linked to the higher level of feather pecking activity observed in the control chicks.

### 4.4. Effect of Brooders on Fearfulness: Differences between Brooder Treatments

Differences in fearfulness between birds from the four brooder treatments were also found although they were minor and not as clear as between brooder and control birds. However, these differences are more difficult to explain. Birds from SM (Small and Movable) were expressing more fearfulness than birds from the other brooder treatments in minimum one parameter of at least one of the tests at each age. If the increased fearfulness of birds from SM is to be explained by the treatment, it appears that only the combination of brooder size and management of height could be attributed as causing the effect. Birds from the other brooder treatment with the smaller brooder size were among the least fearful in the majority of the tests conducted. Also, birds from the other brooder treatment, where the brooder was raised on a regular schedule during the first four days, were inconsistent in their fear responses with them being most fearful in the NO test conducted when they were chicks and least fearful in the open-field test when they were pullets and adults. Thus, neither the size of the brooder nor whether it was raised regularly seems to result in differences in fearfulness, but apparently the combination of a small brooder raised at a regular schedule during the first four days does.

## 5. Conclusions

In conclusion, the present study demonstrates that providing layer chicks with dark brooders during the brooding period results in changes of their time budget in terms of calmer chicks expressing less abnormal behavior during the first six weeks of life and in long-term reduction of the level of fearfulness. The knowledge of the effects of dark brooders gained in the present and previous studies [2,3,8,19,42] provides firm evidence that dark brooders can be a successful method of reducing or preventing some of the major welfare problems in layers, e.g., feather pecking, cannibalism and fear.
